# Advanced Thermochromic Ink System for Medical Blood Simulation

**DOI:** 10.3390/membranes11070520

**Published:** 2021-07-11

**Authors:** Mohammad Noorizadeh, Abdullah Alsalemi, Yahya Alhomsi, Aya Nabil Khalaf Mohamed Sayed, Faycal Bensaali, Nader Meskin, Ali Ait Hssain

**Affiliations:** 1 Department of Electrical Engineering, Qatar University, Doha P.O. Box 2713, Qatar; aa1300250@qu.edu.qa (A.A.); yahia.alhomsi@qu.edu.qa (Y.A.); as1516645@qu.edu.qa (A.N.K.M.S.); f.bensaali@qu.edu.qa (F.B.); nader.meskin@qu.edu.qa (N.M.); 2 Medical Intensive Care Unit, Hamad Medical Corporation, Doha P.O. Box 3050, Qatar; ahssain@hamad.qa

**Keywords:** simulation-based training (SBT), extracorporeal membrane oxygenation (ECMO), high-realism simulation, blood oxygenation, thermochromic ink, blood simulation

## Abstract

Simulators for extracorporeal membrane oxygenation (ECMO) have problems of bulky devices and low-fidelity methodologies. Hence, ongoing efforts for optimizing modern solutions focus on minimizing expenses and blending training with the intensive care unit. This is particularly evident following the coronavirus pandemic, where economic resources have been extensively cut. In this paper, as a part of an ECMO simulator for training management, an advance thermochromic ink system for medical blood simulation is presented. The system was developed and enhanced as a prototype with successful and reversible transitions between dark and bright red blood color to simulate blood oxygenation and deoxygenation in ECMO training sessions.

## 1. Introduction

Extracorporeal membrane oxygenation (ECMO) is arguably the most complicated procedure in the intensive care unit (ICU) [1], and it is an intrusive life-sustaining system that delivers cardiopulmonary support to patients as they recovery from acute respiratory and/or cardiogenic failure [2]. The patients’ critical reliance on ECMO necessitates its continuous operation with minimum errors. Unfortunately, ECMO is fraught with problems exacerbated by the patient’s diagnosis, technical deficiencies of the devices, or professional error and ineptitude of new clinical care staff [3,4]. Due to the susceptibility of ECMO to human mistakes and its technically demanding design, ECMO practitioners must be trained with appropriate scientific, mental, and crisis managing protocols [5]. As ECMO is a high-risk technique, training staff has recently been carried out using simulation-based training (SBT) systems [6,7,8,9].

Until now, the simulation model defined by Anderson has been used by the majority of ECMO centers that provides SBT [10,11]. The scheme is made up of an ECMO circuit loaded with red fluid and attached to a tank with a discreet attachment to a syringe that allows for circuit volume change and air delivery [11]. When the simulation session starts, discrete manual changes are made to the circuit to replicate emergency scenarios [11,12]. Hypovolemia, for example, may be simulated by draining fluid from the circuit when a nurse swings a thread tied to the tubing, causing chatter along the drainage line [11,12].

While practical, using a real circuit and simulator add-ons for SBT has significant disadvantages. In some cases, low-fidelity simulations can distract from the intended scenario, instead of inducing suspension of disbelief. In the event of an oxygenator breakdown, for example, it is impossible to raise the pressure through the oxygenator, generate deoxygenated blood in the return route, or modify blood gas saturations shown on current ECMO consoles or in-line displays without complicated circuit adjustments or the use of actual blood. Many other scenarios have the same issue, which is primarily triggered by the simulator’s inability to monitor circuit and blood parameters, as well as replicate relevant circuit cues. Many consumer ECMO simulators overcome some of these concerns by supplying instructors with a wirelessly operated screen that displays pertinent parameters [13,14]. There is also a disparity between the real parameters shown on the actual ECMO machine and those shown on the emulated displays. Second, removable ECMO circuit elements, such as the oxygenator, are costly, rendering continuous substitution for SBT difficult or impossible [15].

Motivated by the above pitfalls, we are working on a standalone, modular ECMO simulator that does not involve the intervention of a real ECMO computer or its costly circuit components. The central idea is to build an ICU ECMO atmosphere that instructors can completely monitor, and learners can engage with. This is accomplished by developing low-cost, high-fidelity components that can reproduce ECMO indicators in a cost-effective manner [16,17]. Additionally, the contribution of this article is focused on simulating the visual cue of the oxygenator function (i.e., membranes), which is oxygenating blood. Therefore, instead of using real membranes in simulations, which is costly and possibly contaminative, a novel thermochromic ink solution is proposed with a standalone heater–cooler. Therefore, our initial research activities were inevitably focused on simulating the prominent ECMO visual indicator: blood oxygenation color shift. When blood gains oxygen and eliminates carbon dioxide, its color changes from dark-red to red. This is achieved by using thermochromic ink, a special substance that changes its shade of color according to heat. Therefore, to power thermochromic ink in ECMO SBT, a heating (and cooling) control system is required. In this article, we describe the development and testing of a novel heater–cooler thermochromic ink control system for ECMO simulation.

The remainder of this article is structured as follows. Related work is recounted in Section 2. The simulator overall design is described in Section 3. Section 4 depicts the development of the simulated ink heater–cooler module, and Section 5 reports the achieved results of the implemented prototype with performance analysis. The article is then concluded in Section 6.

## 2. Related Work

ECMO hands-on experience is essential for medical personnel to appreciate the sophistication of the treatment and the relationship between the ECMO parameters and the patient. Furthermore, the medical personnel are engrossed in emergency situations through SBT in order to save patients with well-practiced swift interventions and activities working as a team [1]. Since, to the best of the authors’ understanding, there is no comprehensive uniform simulation protocol for ECMO, simulation technology and methodologies vary between training centers.

Despite increasing popularity, especially after the spike of the present coronavirus pandemic (COVID-19) [2,3], advances in ECMO SBT is catching on to the demand [4]. Hence, training experts are forced to utilize available equipment and practices at hand to recreate believable SBT scenarios, as much as possible, which has led to the engendering of some common simulation practices in the literature [5].

Currently, simulated ECMO circuit are developed to provide easy, do-it-yourself (DIY) solutions to replicate some of the patient-related complicated procedures of ECMO. These practices, pioneered by Anderson [6,7], are quite common in many ECMO training centers. On the other hand, researchers employing the inherent synergy between medical sciences and engineering came up with more technologically advanced solutions. As a case study, the Orpheus perfusion simulator was first used with a simulated ECMO circuit, and a modified mannequin with PVC tubing that passes through it [8]. Connected in series between the arterial and venous tubes, the simulator hydraulically replicates the heart, arterial, and venous systems.

Moreover, the NIJMEGEN ECMO Simulator is a portable system that can emulate common parameters and that can be controlled via a wireless interface [9]. Another simulator is the advancement developed by Puślecki et al. [10,11], which is an economical hydraulically controllable mannequin simulator, allowing for pressure simulation and cannulation training.

Once the research caught interest, commercial solution started entering the available ECMO SBT market. The machines are advertised as plug-ins to existing ECMO systems, naturally requiring an ECMO circuit and real blood or dyed liquid, or even water. The EigenFlow and Parallel Simulator are prime examples, which can control common circuit parameters and recreate some scenarios with the privilege of wireless real-time control. Additional parameters are provided, including standard bedside monitors [12,13]. Furthermore, cannulation can be simulated using the ECMO Patient Simulator (EPS) by Biomed [14]. The simulator also aids in training the involved process of connecting the circuit. Additionally, the Medos Deltastream HC heater/cooler provides intelligent temperature control during perfusion and can be used with all oxygenators to provide accurate temperature management for patients. In addition, the ParaTherm heater/cooler for the intensive therapy unit (ITU) or inpatient transportation employs thermo-electric heating and cooling via Peltier tiles, replacing refrigerants or submerged heating devices [12]. Both machines, however, are not ECMO simulators and are not designed for SBT purposes [15].

Despite these positive advancements, key limitations still impede creating a holistic learning experience that does not drain financial resources. Firstly, most of the literature provides decent SBT tools; yet, they are intrinsically expensive because of the reliance on disposable equipment such as oxygenators, heat exchangers, and pumps, which are not only costly, but can be used on actual patients. In the event of pandemics and increased demand, such resources are prioritized for real patients in peril, and not for SBT. Even more, the current processes require a manual adjustment of the ECMO (e.g., circuit alarms), which damages the intended suspension of disbelief. Table 1 compares existing simulators in terms of their features and areas of improvements.

The more striking observation is the inherit need for real blood to carry out the simulation directly, since real equipment is usually used. A typical oxygenator, for example, gets damaged when water or non-blood liquid is passed through it. Hence, the status quo of simulation systems does not provide a suitable means for effective visualization of the primary function of ECMO, which is oxygenation.

In this work, in order to advance upon the reviewed literature and fill the biggest gaps found, a novel ECMO simulator is developed, focused on high-realism as much as cost-effectiveness. Specifically, we focus on the blood simulation part [16], which is responsible for recreating oxygenation, hypoxemia, and recirculation, with a thermochromic ink-enabled heater–cooler module controllable via a tablet application. This is a novel advancement in ECMO SBT that enables pioneering simulation fidelity at very low cost.

The authors of this work have also published about other aspects of the proposed simulator [17,18,19,20]; however, this work elaborates on the heater–cooler system for blood simulation.

## 3. Overview of the Modular ECMO Simulator

**Figure 1:**
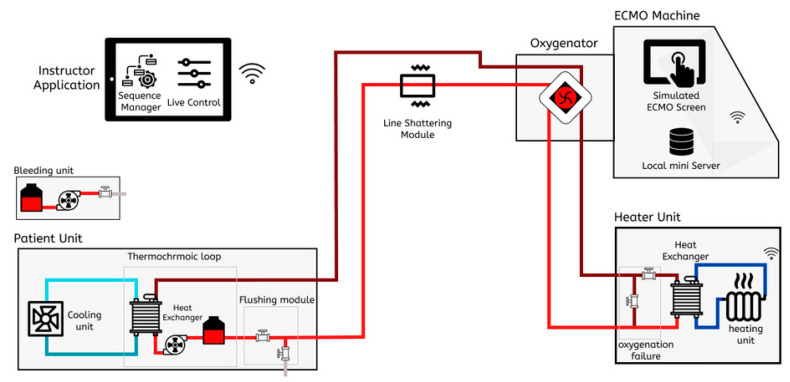
Overview of the ECMO simulation system.

Our approach of creating a modular and low-cost simulator while maintaining a high level of realism is to focus on generating the visual and audio cues of the ECMO producer and operation without the need to use a functional ECMO machine or expensive machinery. To implement such a concept, the simulator is made up of multiple simulation modules, where each module is responsible for creating one visual or audio cue. All of these modules are controlled by an instructor tablet application. This methodology gives the instructor full control of each visual or audio effect that can occur in a real life ECMO circuit. The simulation modules are grouped together in four different units to match the position of each cue in the real life ECMO setup. Figure 1 shows the modular ECMO simulator system setup.

### 3.1. Patient Unit

The patient unit is the core unit of the simulator, where the core simulation module is based along with most of the simulation modules used in the simulation system as it is placed near the patient bed, where most of the visual and audio effects occur.

The main simulation module is responsible for recreating the color change effect that happens in the blood tubes between the ECMO machine and the patient in a real ECMO circuit. The purpose of this simulation module is to constantly change the color between dark red and light red to illustrate blood oxygenation and deoxygenation, which is the main function of the ECMO machine. To achieve color change in a controllable method, we have developed a system based on the thermochromic ink that changes color based on its temperature. As Figure 1 shows, the thermochromic ink flows through two heat exchangers, where the thermochromic ink is heated at one end to change the color of the ink to light red and then cooled to change the color to light red. Applying this method, will allow for the instructor to control the color change of the circuit to simulate a real-life scenario of how the color of the blood in real life changes between dark red and light red. Once the thermochromic loop is implemented, other simulation modules can be designed and implemented to execute the visual and audio effects that occur on the blood tubes.

### 3.2. The ECMO Unit

The ECMO unit takes the shape of the real ECMO machine and color to mimic its appearance to allow the trainee to have the feeling of using the real machine. Inside the ECMO unit a single board computer is used to drive the 5-inch touch screen that shows the control panel of the ECMO circuit and shows values that are related to the patient and the circuit, such as pump rpm, blood pressure, blood temperature, etc. All of these values can be controlled by the instructor application to enable a warning on each individual patient-related value in order for the trainee to take action accordingly and resolve the warning.

### 3.3. The Oxygenator Unit

The oxygenator unit is responsible for providing a path for the thermochromic ink to flow from the patient unit to the heat unit and then back to the patient unit to apply heat to the thermochromic ink inside the heat unit and thus change the color from dark red to light red. In order to provide this path, the oxygenator is recreated using a 3D printer to mimic the real oxygenator shape and color. With such approach, the cost of running a simulation session drops greatly due to the unnecessity of using a medical grade oxygenator, which is very expensive.

### 3.4. The Heater Unit

The heater unit function is to complete the thermochromic loop heating and cooling cycle in order to change the color from dark red to light red. Inside this unit, a heating unit is installed to provide hot water to the heat exchanger that the thermochromic ink flows through. As Figure 1 shows, the instructor can control two valves through the instructor application that will bypass the heating stage to keep the loop color dark red, which indicates a real-life scenario of oxygenation failure. After the trainee performs the required steps in order to solve the oxygenation failure, the instructor can switch off the valves again to allow the thermochromic loop to flow through the heat exchanger and thus allow the color to change.

### 3.5. Preparing Thermochromic Ink

The novel use of thermochromic ink has been patented, employing cost-effective simulation of blood color change. As in indicated in [16,19], preparing a thermochromic ink mixture requires the use of commercial black thermochromic ink that activates at 31 °C, and non-staining yellow and pink dyes mixed in 1 L of distilled water. Further details of its use can be found in the patent application.

## 4. System Design

The system design consists of three main parts: (i) the thermoelectric module, (ii) the cooling unit, and (iii) the heating part.

### 4.1. Description of the Thermoelectric Module

The thermochromic circuit’s core function is color change by increasing/decreasing the temperature to above/below the thermochromic ink deactivation point, as described in Section 3.5. As a result, the heat exchange mechanism is essential to adjust the temperature accordingly. A thermoelectric device is a transducer that can produce electricity by exposing it to heat and vice versa, which is known as the Peltier effect. Heat, therefore, can be induced by passing electric current through the thermoelectric module, passing through two separate semiconductors, resulting in the generation of heat or cold. In other terms, a thermoelectric device has two sides; as one of them cools, the other face heats up. Furthermore, the efficiency of the cooling side is directly linked to the performance of the heating side, which implies that by lowering the temperature of the warming side, the performance of the cooling side will greatly improve.

### 4.2. The Cooling Part

**Figure 2:**
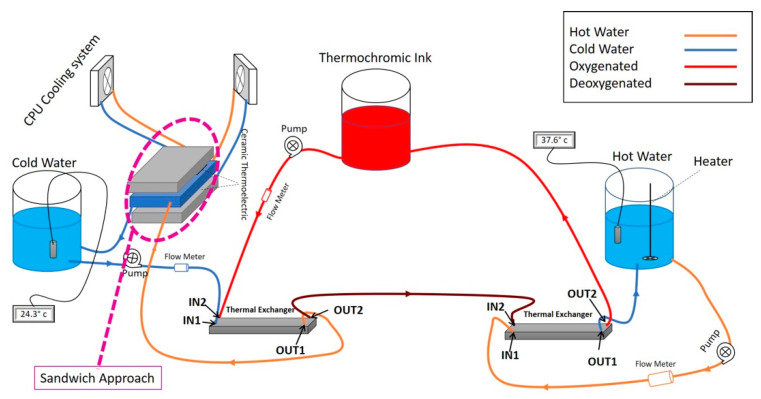
Overview of the heater–cooler prototype.

As illustrated in Figure 2, the cooling unit is comprised of a number of thermal exchangers, ceramic thermoelectric modules coupled with two CPU cooling units. Liquid is stored in a tank and passes through pipes. Fluid goes through aluminum blocks circulated by a pump (PMP-300, Koolance/Auburn/WA/USA), where the fluid’s flow and temperature are measured by a flow metering device (INS-FM14, Koolance/Auburn/WA/USA) along with a temperature sensor, respectively.

The thermal exchanger has four terminals, as seen in Figure 2: IN1, IN2, OUT1, and OUT2. As a result, thermochromic ink is supplied to IN1 and exits from OUT1. Furthermore, the cooling unit affects the thermochromic ink that enters IN2 and then exits by OUT2. Indeed, by affixing two thermoelectric devices to the aluminum water/coolant cooling device, the temperature of the water/coolant contained inside this block can be reduced. It would also flow between the cooling tank and the thermal exchanger device through a controllable pump. Two CPU cooling units, on the other hand, lower the temperature of the thermoelectric module’s heating side. To ensure compatibility, the unit components are chosen from the same vendor. Furthermore, since flowrate has a significant impact on cooling efficiency, several flowrate meters have been installed in the circuit after each pump. In other words, increasing the flowrate reduces the cooling impact of the cooling device significantly. Flowrate, on the other hand, is a fundamental element in this prototype, and it is not negotiable. As a result, by determining the best trade-off between flowrate and temperature, overall efficiency can be improved. Furthermore, in order to improve the performance of the cooling unit, the Sandwich technique is used.

#### Sandwich Method

**Figure 3:**
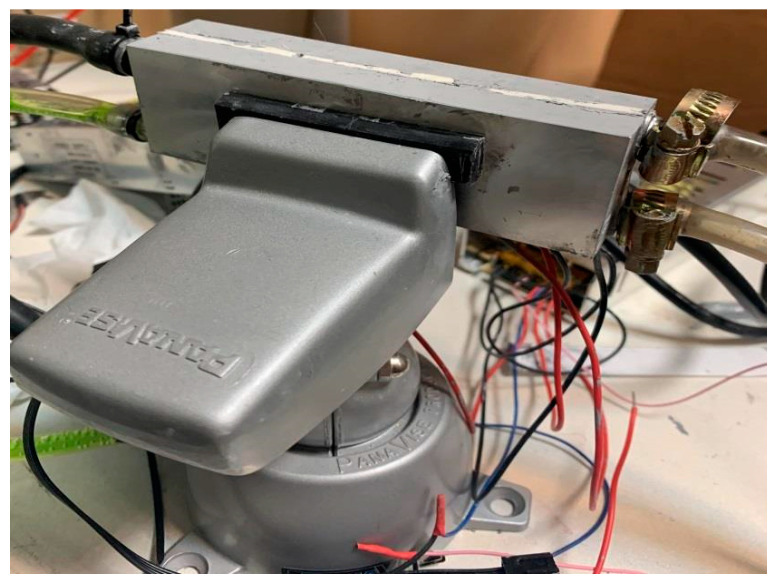
The aluminum block is attached to the thermoelectric modules from one side only.

**Figure 4:**
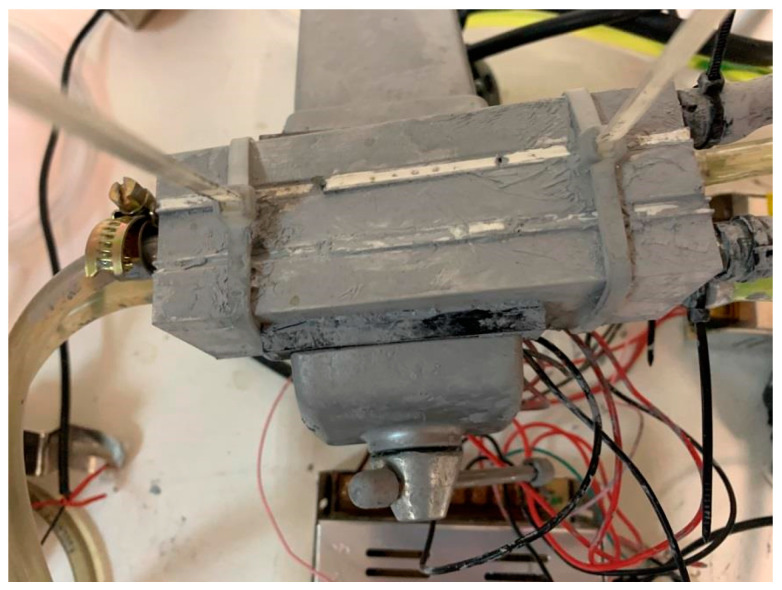
The aluminum blocks are attached to the thermoelectric modules from both sides of cooling block.

By performing a trial-and-error technique, and due to existing challenges, such as high flowrate, large amounts of thermochromic ink, and heating effects from the heater side, the sandwich approach is applied. In other words, as shown in Figure 3, the initial test is performed by using three thermoelectric modules and two aluminum cases in a way that one aluminum case contains the cold water/coolant and the other one carries the dissipated heat from the thermoelectric module. However, because of existing challenges such as the effect of the heating unit on the cooling unit and high transient time for reducing the temperature to the desired value, we added three more thermoelectric modules and one more aluminum block. Thus, as depicted in Figure 4, the aluminum case, which contains the cold water, is bounded with six thermoelectric modules, and two aluminum cases in both side of the cold aluminum case control (cooling down) the heat generated by the thermoelectric modules.

### 4.3. The Heating Part

**Figure 5:**
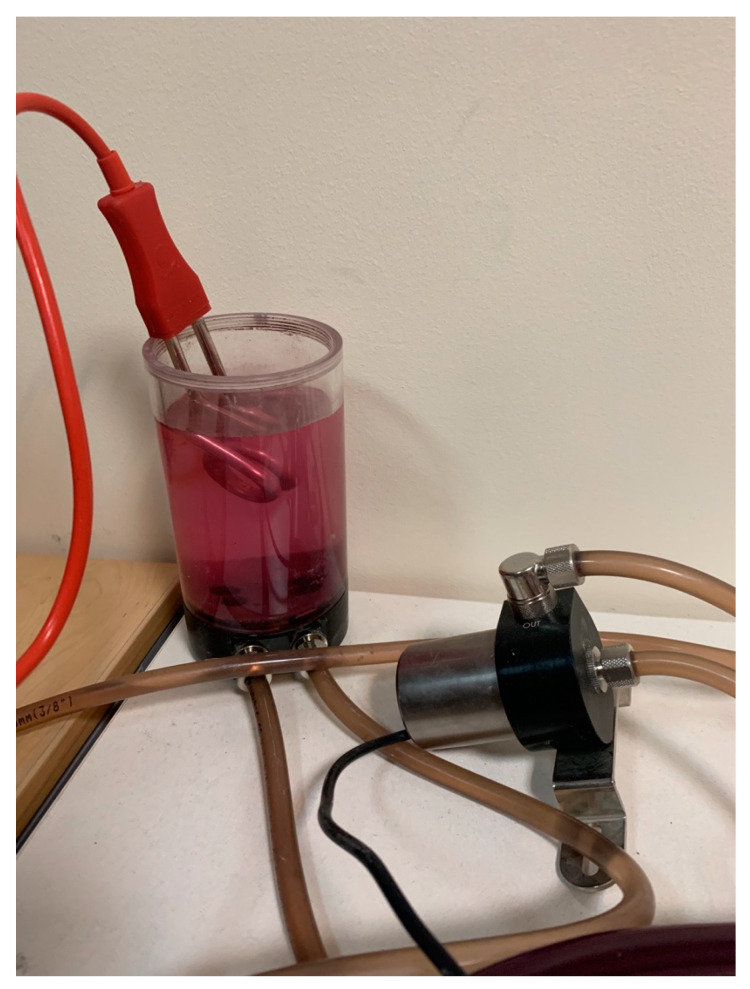
The heater element.

As depict in Figure 5, the heating unit consists of a number of thermal exchangers and a heating element. Liquid is stored in a tank and passes through pipes. Fluid goes through the aluminum block circulated by a pump (Koolance PMP-300), where the fluid’s flow and temperature are measured by a flow metering device (Koolance INS-FM14) along with a temperature sensor, respectively.

The heating unit includes almost the same cyclic process as the cooling unit. However, in order to heat up the tank’s water, a heating element is used. Hence, as shown in Figure 5, by placing the heater inside the water tank, the water’s temperature increases.

## 5. Results and Discussion

**Figure 6:**
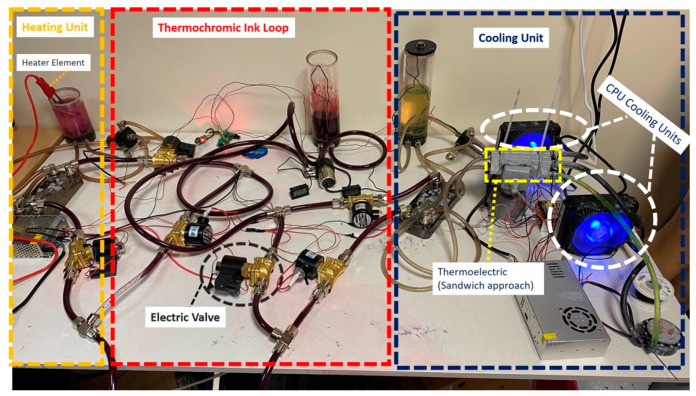
Overview of an enhanced version of the heater–cooler prototype (normal/oxygenation operation).

**Figure 7:**
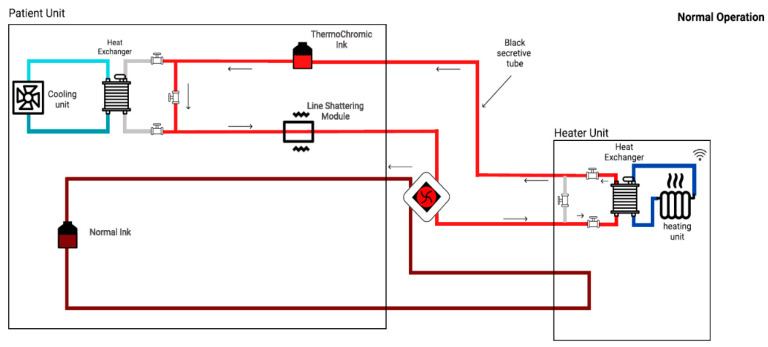
Oxygenation process.

**Figure 8:**
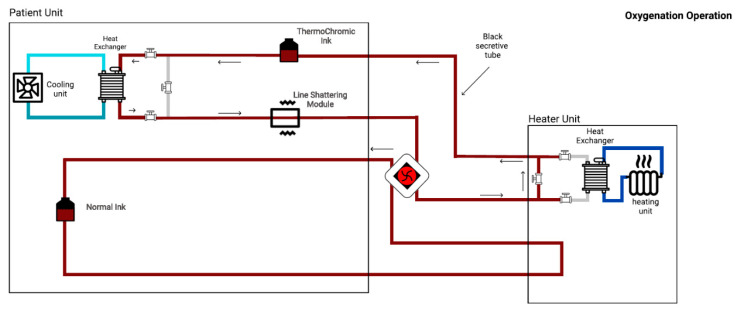
De-oxygenation process.

Indeed, the thermochromic ink’s tank contained the bright ink during the simulation session. Hence, the ink’s color turned to dark red once the ink was injected to the cooling unit. Likewise, as shown in Figure 6, the ink transferred to the heater unit to turn the color back to bright red. Furthermore, in order to compensate the non-linear terms such as flow-rate, effect of heat-transfer, and others, the bypass technique was used. As depicted in Figure 7 and Figure 8, three valves were added at each heat exchanger in order to bypass the loop. Subsequently, by isolating heater and cooler units from each other, the effect of heat-transfer to each loop was minimized. Therefore, the prototype’s response time to color change decreased (i.e., faster response). Moreover, an additional tank that always contains the dark ink was added to the system. As demonstrated in Figure 7 and Figure 8, the additional tank was added to enable the oxygenation and deoxygenation scenarios in a faster time frame. Consequently, several scenarios could be obtained in a shorter period.

In order to test and validate the prototype, several parameters needed to be monitored: temperature of each tank, flow rate of each pump, and visible color-change. Moreover, the successful test can be defined as follows:

- Complete integration with the patient unit;
- Seamless wireless communication with the instructor’s tablet;
- Attain required ∆T in specific time frame for thermochromic ink.

Subsequently, two scenarios were defined in order to test the prototype: (i) oxygenation; and (ii) de-oxygenation.

As depicted in Figure 7, in the oxygenation scenario, the dark thermochromic ink (cold) will go to the heater unit to increase the brightness of the thermochromic ink. In other words, the de-oxygenated (dark color) blood will go to the oxygenator in order to oxygenate the blood (bright color). Indeed, the thermochromic ink’s temperature needs to increase up to 37 °C and will take up to 3 min to reach that temperature value.

As shown in Figure 8, the de-oxygenation scenario will occur in the absence of the heater unit (or oxygenator). Consequently, all the pipes will turn to the dark red color. In fact, the thermochromic ink’s temperature needs to drop to 25 °C. Consequently, by enhancing the cooling unit, the temperature can be dropped to 25 °C (from 37 °C) in 4–6 min.

Subsequently, the entire liquid is circulating via those three pumps. In order to improve the performance of the prototype and due to the proportional effect of flow to the performance of the heater/cooler, three flowrate sensors were implemented in the circuit in order to control the flowrate and, eventually, to improve the entire process to operate autonomously. Likewise, a power supply is also provided in order to supply power to the pumps, CPU cooling systems, heater, flowrate meters, and the thermoelectric modules. Furthermore, Table 2 shows the cost analysis of the developed system, with an estimated overall cost of the developed system of about USD 700.

### 5.1. Instructor App

**Figure 9:**
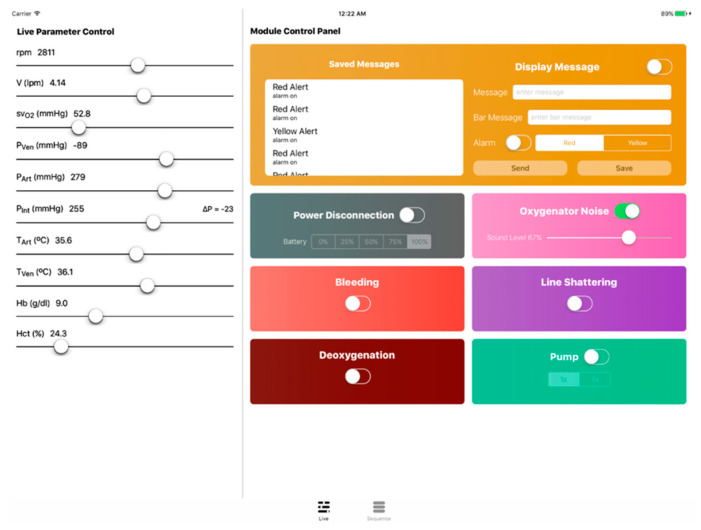
The instructor app including the live control panel view.

**Figure 10:**
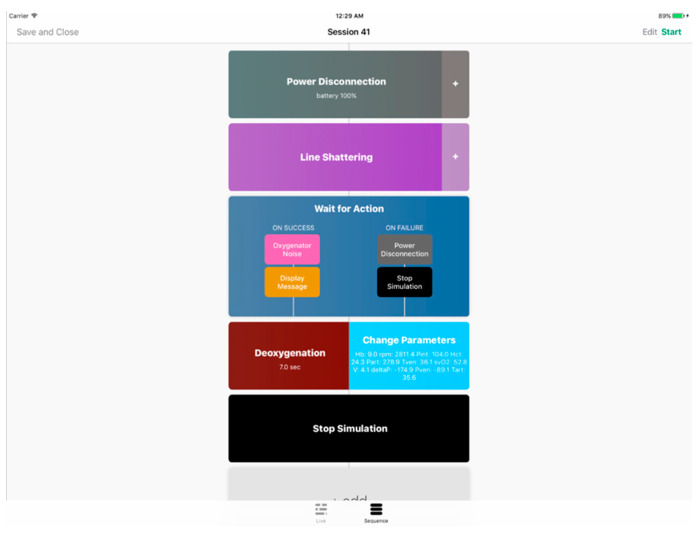
The instructor app showing the sequence manager view.

As was previously mentioned, a tablet application was developed to operate the simulator and conduct emergency scenarios. The instructor app features a live control panel as well as a sequence manager that is connected to the cloud for parameter and sequence transmission. Over at the simulator’s main unit, a python client program receives commands (e.g., parameter adjustments and scenario files) and operates the various modules accordingly. The instructor app shown in Figure 9 and Figure 10 was developed in Swift for iOS and installed on an iPad.

To elaborate on the instructor app features, firstly, through the live control panel, the ECMO instructors are provided with complete manual control of the several simulator components. It showcases live ECMO control over many parameters (e.g., bleeding unit, line shattering, power disconnection, oxygenator noise, deoxygenation, etc.). By including this feature, a spontaneous simulation is allowed, eliminating the need to conduct an unnecessary full emergency scenario. Secondly, ECMO instructors may use the sequence manager to create a variety of tailored emergency training scenarios. Software simulation modules are arranged on a timeline to produce a training sequence. Modules are divided into two categories: generic and emergency. Emergency modules imitate genuine ECMO circuit impediments, whereas generic modules implement structural functionality to an ECMO scenario.

### 5.2. ECMO Simulation Possibilities

High-fidelity simulation sessions help learners use their imaginations and avoid rudimentary and expensive alternatives, which is essential for effective SBT. High-fidelity technologies and environments, on the other hand, are expensive and necessitate a large financial investment. Incumbent commercial solutions, as shown in the literature review, are costly and necessitate a significant financial investment. In addition to simulation facilities, ECMO centers that include SBT depend on a working ECMO system as well as costly consumable circuit modules that provide little background, since the visual/audio cues they generate and parameters they present are usually unreliable or uncontrollable in relation to implemented scenarios [19].

Using the proposed method, a variety of critical scenarios can be recreated using a simulated ECMO circuit with the special thermochromic effect that mimics the color shift of blood oxygenation in actual ECMO circuits. The circuit employs a controlled and continuous heat exchange operation, resulting in a temperature difference between the drainage and return lines and, as a result, a color difference due to thermochromism. It may generate visual cues for a variety of emergency situations, such as oxygenator loss, disconnected gas supply, elevated oxygen intake, reduced lung capacity, insufficient circuit flow, and recirculation. ECMO experts assessed the fidelity of the thermochromic impact and determined it to be realistic.

**Figure 11:**
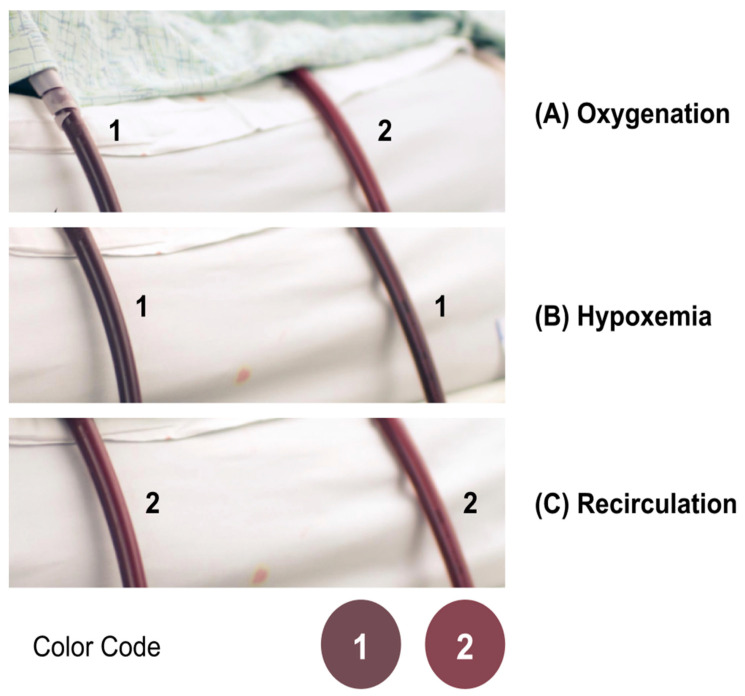
Simulating (**A**) oxygenation, (**B**) deoxygenation, and (**C**) recirculation using a simulated ECMO circuit.

When blood gains oxygen (O2) and lacks carbon dioxide (CO_2_), its color changes from dark-red to red. It is an important diagnostic tool that signals regular activity, active ECMO activation, low oxygen saturation in the return line, and recirculation. As shown in Figure 11, oxygenation, deoxygenation (hypoxemia), and recirculation is successfully simulated using the thermochromic system.

Moreover, the developed system is considered the simulation foundation for recreating circuit-side complications such as hypovolemia, air embolism, flow occlusion, and more. By the of thermochromic ink, multiple scenarios can be enabled in a highly-realistic and cost-efficient manner [17,18].

## 6. Conclusions

In this paper, a technological advancement for ECMO simulation is presented, namely a standalone, cost-effective heater–cooler system for blood simulation with the aid of thermochromic ink, a special ink that is used to simulate oxygenation, deoxygenation, and recirculation without the use of real blood. The design of the system involves the use of thermoelectric modules and CPU coolers hand in hand with the patient unit thermochromic loop, optimizing for maximum efficiency and cost effectiveness. After system design and prototyping, testing and validation are carried out to evaluate the performance and identify aspects of improvements. Integrated with a wireless instructor application, the current prototype can provide on-demand efficient heating and cooling functionality in a timely manner. After addressing the limitations of the lack of closed-loop control, bulkiness, and using a minimum amount of ink, the system will be integrated with the simulator, allowing new simulation possibilities to fight COVID-19 and beyond.

## 7. Patents

The following US/PCT patent applications have been filed: A. Alsalemi et al., “Using thermochromic ink for blood simulation in medical training,” US20190251869A1, 15 August 2019 and A. Alsalemi et al., “Using thermochromic ink for blood simulation in medical training,” WO2019159051A2, 22 August 2019.

## Figures and Tables

**Table 1:** Comparison of existing ECMO simulators.

Simulator Creator	Type	Features	Areas of Improvements
Lansdowne et al. [8]	Academic	Normal procedure simulation	Real equipment needed Expensive oxygenation simulation
NIJMEGEN [9]	Commercial	Parameter emulation Portable Customizable Wireless interface	Real equipment needed No standard simulation scenarios No blood simulation
Puślecki et al. [10,11]	Academic	Hydraulic pressure simulation Can simulate cannulation Economical	Real equipment needed No wireless control
Curtis Life Research [13]	Commercial	Can simulate cannulation Wireless interface	Real equipment needed No standard simulation scenarios Limited settings
Biomed [14]	Commercial	Parameter emulation Custom scenarios	Real equipment needed
Alsalemi et al. (this work) [20]	Academic	Blood simulation Economical Custom and standard scenarios Wireless interface	Needs replacing thermochromic blood at every 12 h
3Dmed [21]	Commercial	Artificial blood Can simulate cannulation Can circuit connection	Real equipment needed Few standard simulation scenarios No wireless control

**Table 2:** Cost analysis of the developed system.

Component	Unit	Estimated Price per Unit (USD)
Tank	3	20
Pump	3	40
Flow-meter	3	20
Temperature sensor	3	11
Aluminum block	3	10
Thermoelectric	6	3
CPU cooling unit	2	75
Heating element	1	3
Power supply	2	20
Heat-exchanger	2	80
